# Inversion of Soil Parameters and Deformation Prediction for Deep Excavation Based on PSO-SVM Model

**DOI:** 10.3390/s25206281

**Published:** 2025-10-10

**Authors:** Jing Zhao, Longhui Chen, Hongyin Yang, Bin Li, Linlong Yang, Hao Peng, Hongyou Cao

**Affiliations:** 1 China Railway 11th Bureau Group First Engineering Co., Ltd., Xiangyang 441199, China; 2 School of Civil Engineering and Architecture, Wuhan Institute of Technology, Wuhan 430073, China; 3 Capital Construction Department, Wuhan Textile University, Wuhan 430073, China; 4 School of Civil Engineering and Architecture, Wuhan University of Technology, Wuhan 430070, China

**Keywords:** deep excavation, PSO-SVM model, soil parameters inversion, finite element simulation, deformation prediction

## Abstract

During deep excavation, actual soil parameters undergo changes. To enhance the accuracy of soil parameter selection in finite element simulation and improve the precision of finite element analysis, an inversion method for soil parameters based on a PSO-SVM model is proposed. In this method, the particle swarm optimization (PSO) algorithm is utilized to optimize the penalty parameter *C* and kernel function parameter *g* of the support vector machine (SVM) model. The optimized PSO-SVM model is employed to establish a nonlinear mapping relationship between the horizontal displacements of retaining structures in deep excavations and soil parameters through orthogonal experimental design and finite element simulation analysis. Subsequently, soil parameters are inverted from monitoring data of horizontal displacements of retaining structures, and the reliability of the parameters is verified. The deformation of the retaining structures during subsequent cases is then predicted. The results demonstrate that the absolute error of the peak maximum horizontal displacements of the retaining structures after inversion is maintained within 1 mm. The maximum relative error is reduced from 18.96% before inversion to 7.63%, indicating that the inverted soil parameters for the deep excavation possess high accuracy. The precision of the finite element simulation for deep excavation is significantly improved, effectively reflecting the actual mechanical properties of the soil during the construction stage. The inverted parameters can be used for the prediction of subsequent retaining structure deformation. During subsequent construction conditions, the predicted maximum horizontal displacement (deformation) of the retaining structure at monitoring point CX1 is 15.66 mm, and that at monitoring point CX2 is predicted to be 14.22 mm. Neither value exceeds the project warning threshold of 30.00 mm.

## 1. Introduction

With rapid economic development and urbanization in China, many cities are faced with problems such as land-resource shortage [1,2], traffic congestion [3,4], and insufficient greening [5,6]. Consequently, the development and utilization of underground space are increased accordingly [7,8,9]. Especially in the construction of large subway stations and urban road tunnels, deep excavation engineering is recognized as an indispensable and critical component [10]. The deep excavation construction process is considered a continuous change process. During the construction stage, the process may be affected by unavoidable factors such as rainfall- and construction-induced deformation [11,12], which cause actual soil parameters to differ from those determined by site investigation [13]. Therefore, how to accurately reflect the actual mechanical parameters of the soil during the construction process has become a key issue in the research on soil and retaining structure deformation within the context of underground-space development [14,15].

In recent years, intelligent methods such as neural networks [16,17] and evolutionary algorithms [18,19] have been widely applied to parameter inversion tasks due to their strong nonlinear capabilities. Within deep excavation engineering, the inversion of soil parameters based on monitoring data has become a research focus [20,21,22,23]. This approach enhances the accuracy of numerical simulations and enables the precise prediction of excavation deformations. PSO combined with a back propagation (BP) neural network was investigated by Li, Qingwang et al. [24]. Excavation deformations were simulated using PLAXIS. Soil layer parameters were then inversely derived through monitoring data, enabling dynamic prediction and error reduction. Methods including wavelet transform, convolutional neural networks (CNNs), and long short-term memory (LSTM) networks were integrated by Wei, Xing et al. [25]. Soil–water coupling parameters influencing the horizontal displacement of deep excavations were inverted using an artificial neural network model, achieving high-precision prediction. The fundamental principles of machine learning models, including networks, genetic algorithms, and clonal-selection algorithms, were elaborated by Wang, Xiang et al. [26] in predicting deep excavation supporting structure deformation and surrounding surface settlement and assessing foundation pit risks. The accuracy and efficiency of these methods for displacement inversion in deep excavations were emphasized. A GWO-MLSSVR model was proposed by Chen Changrui et al. [27] for the inversion of hardening soil parameters for fill layers. Validation was performed using a case study of a deep excavation in Xiamen, yielding significantly higher accuracy compared with traditional methods. Although the aforementioned studies have achieved significant results in parameter inversion, their primary focus remains on the inversion of monitoring data. Nevertheless, the ultimate goal of parameter inversion is to achieve accurate predictions of future deformations, thereby supporting proactive construction-risk management. Currently, research specifically addressing the use of inverted parameters to predict deformations in subsequent construction cases remains relatively limited. Thus, after achieving accurate parameter inversion, this study further predicts future construction conditions to enable dynamic monitoring.

The SVM model exhibits a strong capability in small-sample learning, low classification and prediction errors, and an excellent generalization ability, effectively avoiding overfitting. It can map nonlinear problems from a low-dimensional space to a high-dimensional space where they become linearly separable, making it highly suitable for addressing complex nonlinear relationships in data. The PSO algorithm possesses excellent global search capability, high efficiency, and fast convergence. Compared with other algorithms, PSO is conceptually simple and involves fewer parameters. To enhance the classification performance of the SVM model, the PSO algorithm, with its superior global search ability, is integrated with the SVM model to optimize its penalty parameter *C* and kernel function parameter *g*, thereby constructing an efficient and highly accurate parameter inversion model.

The SVM model is widely applied in many fields [28,29]. In structural damage identification, modal frequencies and mode shape features were classified into different damage levels by the SVM model through the construction of an optimal hyperplane by Thanh Cuong-Le et al. [30]. Verification results showed that the PSO-SVM model achieved high accuracy in low-damage cases and outperformed traditional machine learning methods. In deep foundation excavation, high precision prediction has been difficult to achieve by traditional models due to the complexity of influencing factors and the limitation of data samples. The SVM model has been regarded as an effective method for solving engineering problems because of its strong capability in small-sample learning, low classification and prediction errors, and excellent generalization ability [31]. Meanwhile, its parameter selection and prediction accuracy have been further improved through combination with algorithms such as PSO. The method has been applied in deformation prediction such as displacement and settlement during deep-foundation pit construction.

A soil parameter inversion method that combines particle swarm optimization (PSO) and support vector machine (SVM) is proposed in this study. The method improves the accuracy of soil parameter selection influenced by environmental conditions in deep excavation. The precision of finite element simulation is also improved. The method first establishes a theoretical framework. The soil parameters of the SVM model are optimized by the PSO algorithm. The optimized SVM model is used to build a nonlinear mapping between the horizontal displacement of the retaining structure and the soil parameters. An engineering case study is carried out. A detailed finite element model is developed in ABAQUS. A sample set is generated through orthogonal experimental design. A nonlinear prediction model is then constructed. The soil parameters are identified based on monitoring data of the horizontal displacement of the retaining structure. Their reliability is verified. The results show that the proposed method improves the accuracy of soil parameter inversion. The computational precision and engineering applicability of the finite element simulation are also enhanced.

## 2. Soil Parameters Inversion Method Based on PSO-SVM Model

### 2.1. Support Vector Machine Model

Support vector regression (SVR) is the application of the SVM model in regression analysis. [32]. Its fundamental idea is to find a regression function f(x)=〈w,ϕ(x)〉+b that minimizes deviation from the training samples while ensuring flatness of the function. SVR introduces an insensitive loss function *ε*, meaning that if the deviation between the predicted value *f*(*x*) and the true value *y* does not exceed *ε*, the prediction loss is considered to be zero.

The optimization problem of SVR can be formulated as follows:

(1) $$\begin{array}{ll} \min_{w,b,\xi_{i},\xi_{i}^{*}} & \frac{1}{2}\|w{\|}^{2}+C\sum_{i=1}^{n}\left(\xi_{i}+\xi_{i}^{*}\right)\\ s.t. & y_{i}-\langle w,\phi (x_{i})\rangle -b\leq \varepsilon +\xi_{i},\\ & \langle w,\phi (x_{i})\rangle +b-y_{i}\leq \varepsilon +\xi_{i}^{*},\\ & \xi_{i},\xi_{i}^{*}\geq 0,\;\;\;\;i=1,\ldots ,n. \end{array}$$

where *w* is the normal vector of the hyperplane; *b* is the displacement; $x_{i}$ is the feature vector of the *i*th sample type; $y_{i}$ is the label of *i*th sample type; *ε* is the insensitive loss function; and $\xi_{i},\xi_{i}^{*}$ is the slack variables.

After obtaining the optimal Lagrange multipliers $\alpha_{i},\alpha_{i}^{*},i=1,\ldots ,n$ by solving the dual problem, the regression prediction function is as follows:

(2) $$f(x)=\sum_{i=1}^{n}\left(\alpha_{i}-\alpha_{i}^{*})K(x_{i},x\right)+b$$

where $K(x,x_{i})$ is the kernel function.

A significant influence on inversion results is exerted by the kernel function form of the SVM model. For nonlinearly separable data, training sample data are mapped to a high-dimensional space by the kernel function, which enables the data to be linearly separable in this space. Therefore, an input–output nonlinear mapping relationship is established [33]. A strong generalization ability is possessed by the SVM model; the model is primarily composed of an input-feature mapping function, the penalty parameter *C*, and the kernel function parameter *g*. The penalty parameter *C* controls the tolerance degree of the model to training errors, and the kernel function parameter *g* determines the nonlinear characteristics of the mapping function. These two parameters directly affect model performance.

The radial basis function (RBF) kernel is selected as the kernel function for the SVM model due to its excellent capability to capture the local features of samples and strong interpolation ability. Its mathematical expression is given by the following:

(3) $$K(x_{i},x_{j})=\exp\left(-g\|x_{i}-x_{j}{\|}^{2}\right)$$

where *g* is the gamma parameter in the kernel function, which can significantly affect the kernel function.

The performance of the SVM model is significantly affected by the penalty parameter *C* and the kernel function parameter *g*, as shown in the above derivation. To enhance the performance of the SVM model, the PSO algorithm is selected in this research for the optimization of these two parameters.

### 2.2. Particle Swarm Optimization Algorithm

The PSO algorithm is recognized as a reliable machine-learning-model-tuning algorithm. Inspired by the collaborative foraging process of birds, it is characterized by rapid convergence and simple parameter settings [34]. In the PSO algorithm, the fitness of each particle in the population is utilized to evaluate particle quality. Each particle contains information on all randomly initialized penalty parameters *C* and kernel function parameters *g* in the SVM model. The characteristics of an individual particle are represented by its position, velocity, and fitness. The optimal penalty parameter *C* and kernel function parameter *g* for the SVM model are achieved as particles tracking the individual best position (*pbest*) and global best position (*gbest*) [35]. In a *n* dimensional feasible solution space, a particle swarm consisting of *m* particles is assumed. The velocity and position of the *i*th particle are updated by the following equations: [36].

(4) $$\left\{\begin{array}{l} V_{ij}^{k+1}=\omega V_{ij}^{k}+c_{1}r_{1}(P_{ij}^{k}-X_{ij}^{k})+c_{2}r_{2}(P_{gj}^{k}-X_{ij}^{k})\\ X_{ij}^{k+1}=X_{ij}^{k}+V_{ij}^{k+1} \end{array}\right.$$

where $X_{ik}=(X_{i1}^{k},X_{i2}^{k},\ldots ,X_{in}^{k})$ is the *k*th iteration position of the *i*th particle; $V_{i}^{k}=\{V_{i1}^{k},V_{i2}^{k},\ldots ,V_{in}^{k}\}$ is the speed of the *i*th particle; $P_{i}^{k}=\{P_{i1}^{k},P_{i2}^{k},\ldots ,P_{in}^{k}\}$ is the optimal position of the particle; $P_{g}=\{P_{g1},P_{g2},\ldots ,P_{gn}\}$ is the population optimal position; j is the component of the *i*th particle in dimension j; $c_{1}$ and $c_{2}$ are the learning factors; $r_{1}$ and $r_{2}$ are uniformly distributed random numbers between 0 and 1; and ω is the inertia weight.

### 2.3. Soil Parameters Inversion Process Based on PSO-SVM Model

**Figure 1:**
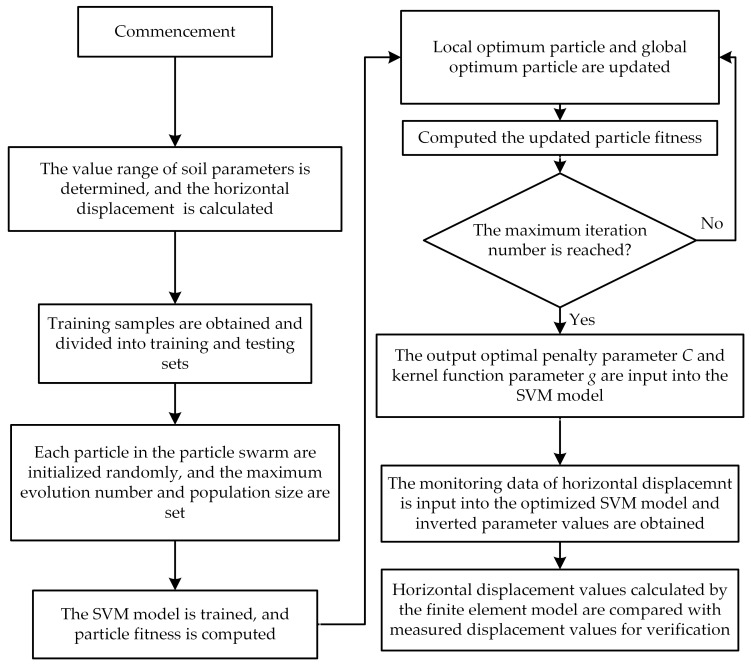
Flowchart of soil parameters inversion process based on PSO-SVM model.

A fundamental procedure for the soil parameter inversion process based on the PSO-SVM model is established with the finite element software ABAQUS (2022) and mathematical software MATLAB (R2024a); the flowchart is illustrated in Figure 1.

1. The value range of soil parameters is determined. Soil parameter samples are constructed based on the orthogonal experimental design. Each set of soil parameters is input into the ABAQUS finite element software to calculate the horizontal displacements of the deep excavation retaining structures. Training samples are obtained and divided into training and testing sets.
2. Using the MATLAB software, the velocity and position of each particle in the particle swarm are initialized randomly. The learning factor $c_{1}$ is set to 1.50 and $c_{2}$ to 1.70. The maximum evolution number is configured as 100. The termination iteration number is assigned a value of 100. The maximum population size M is designated as 10. The penalty parameter *C* and kernel function parameter *g* are constrained within the interval [0.1, 100].
3. The particle fitness of the SVM model under different parameter combinations is evaluated by the PSO algorithm using five-fold cross-validation on the training set (22 groups), The separate testing set (five groups) is strictly reserved for the final evaluation of the model’s generalization performance. When the global optimum is superior to the fitness of the particle itself, the velocity and position of the local optimum particle and the global optimum particle are updated. The updated particle fitness is computed by the SVM model.
4. Whether the maximum iteration number is reached is checked. If “No”, the process is returned to Step (3). If “Yes”, the optimal penalty parameter *C* and kernel function parameter *g* are output.
5. The output optimal penalty parameter *C* and kernel function parameter *g* are input into the SVM model. A nonlinear relationship between the horizontal displacements of deep excavation retaining structures and soil parameters is established using the input sample sets. An optimized SVM model is obtained.
6. The monitoring data of the horizontal displacements of deep excavation retaining structures are input into the optimized SVM model, and inverted parameter values are obtained. The inverted soil parameters are brought into the ABAQUS finite element software. The horizontal displacement values of the retaining structures after inversion are calculated and compared with the monitorings for verification.

## 3. Soil Parameters Inversion Based on PSO-SVM Model

### 3.1. Deep Excavation and Monitoring Points

**Figure 2:**
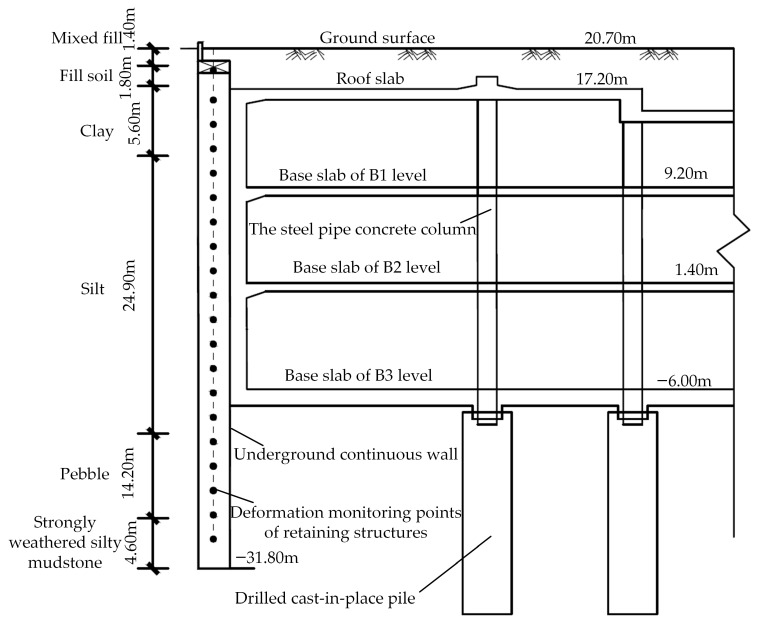
The standard cross-section and geological profile of the deep excavation.

The Wuhan Zhongyi Road Metro Station is constructed as a three-story underground island station. It covers an area of 65,903.50 m^2^, with an external length of 502.60 m, a standard section width of 49.50 m, and a depth of 28.79 m. The maximum excavation depth is recorded at approximately 31.90 m. The shallow excavation is executed by the cut and cover method, while the lower soil and main structure are constructed using the cover-excavation reverse construction method. The main retaining structure is composed of 1.50 m thick underground continuous walls extending 52.50 m deep into bedrock. The internal horizontal support system is designed to integrate with the main structural slabs. Vertical support is provided by 2.50 m diameter drilled cast-in-place piles and permanent 0.90 m diameter steel pipe concrete columns. The excavation site is located in the Yangtze River first-level terrace, with ground elevation in the range of 20.00 m to 21.00 m. The standard cross-section and geological profile of the deep excavation are shown in Figure 2.

The horizontal displacements of the retaining structures are monitored by automated monitoring instruments. The automated monitoring instruments is provided by Kingmach Measurement and Monitoring Technology Co., Ltd. in Changsha, Hunan Province, China. The automated monitoring instruments consist of a multi-point series omnidirectional displacement meter (JMQJ-7915ATS) and acquisition modules (JMZX-4QH). The multi-point series omnidirectional displacement meter is designed based on multi-array MEMS tilt sensors, with multiple units electrically connected via a single cable along the borehole. The acquisition module collects measurement data, enabling automated collection, transmission, and early warning analysis of horizontal displacement data for the deep excavation retaining structures. Data are acquired by the automated monitoring instruments at a frequency of one per 10 min as configured. Simultaneously, monitoring data are transmitted to the cloud platform.

**Figure 3:**
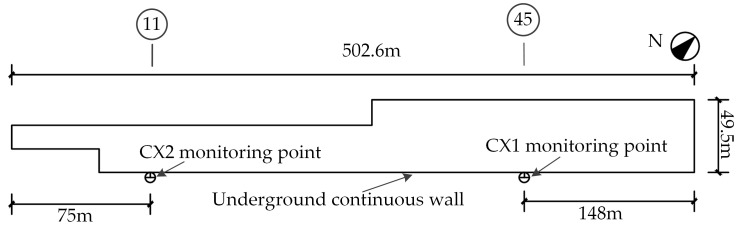
The locations of monitoring points of CX1 and CX2.

**Figure 4:**
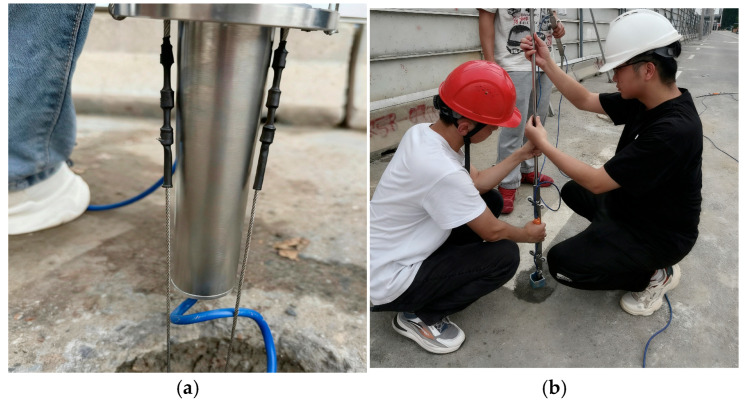
The automated monitoring acquisition sensor and equipment installation: (**a**) the automated monitoring acquisition sensor; (**b**) the installation of automated monitoring equipment.

At monitoring point CX2 located at the south side of the hazardous section (Axis 11) and monitoring point CX1 at the south side of the standard section (Axis 45), The automated monitoring instruments for horizontal displacements of the retaining structures are installed. Deformation data of the retaining structures are collected in real time. Deformation towards the inside of the foundation pit is positive, and deformation towards the outside of the foundation pit is negative. The locations of monitoring points CX1 and CX2 are shown in Figure 3. The automated monitoring acquisition sensor and equipment installation are illustrated in Figure 4.

**Figure 5:**
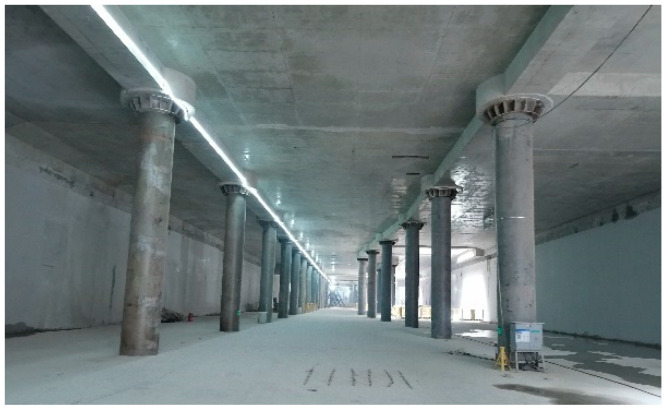
The current status of construction completed at B1 level of the deep excavation.

Based on the construction plan, three main structural construction cases are investigated. Case 1: Excavation of soil at the B1 level, with construction of the B1-level structure and slab. Case 2: Excavation of soil at the B2 level, with construction of the B2-level structure and slab. Case 3: Excavation of soil at the B3 level, with construction of the B3 main structure and base slab. Case 1 has been completed. Case 2 is scheduled for imminent execution. Therefore, monitoring data from Case 1 are utilized for research analysis in this study. Subsequent predictions and analyses of horizontal displacements of the deep excavation retaining structures will be conducted for Cases 2 and 3. The current status of construction completed at the B1 level of the deep excavation is shown in Figure 5.

### 3.2. Establishment of Finite Element Model for Deep Excavation

**Figure 6:**
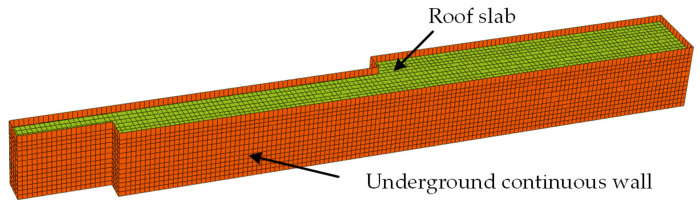
Finite element model of deep excavation retaining structure.

Analysis is performed with the ABAQUS finite element software. According to the influence range of the excavation, the dimensions of the soil finite element model are determined as 690 m × 210 m × 75 m. The mesh consists of 279,466 elements and 303,539 nodes. The Mohr–Coulomb constitutive model is selected as the material model. A gravitational load is applied to the entire model, with a fixed boundary condition imposed at the bottom and a free boundary condition assigned at the top. Normal displacement constraints are applied to the four lateral boundaries. A refined mesh is arranged near the excavation edges, while a coarser mesh is adopted in distant regions. The retaining structures are modeled with C30 concrete. The drilled cast-in-place piles are assigned C35 concrete. All floor slabs are designated C20 concrete. The steel pipe concrete columns are filled with C50 concrete. The soil is simulated with solid elements, while the columns and their pile foundations are represented by beam elements. Other finite element model parameters are detailed in Reference [37]. The finite element model of the deep excavation retaining structures is shown in Figure 6, and the soil parameters are listed in Table 1.

### 3.3. Establishment of Initial Finite Element Model for Deep Excavation

**Figure 7:**
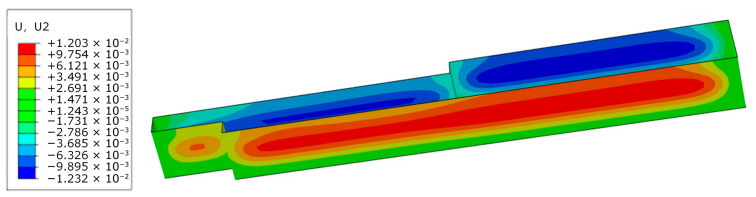
The cloud diagram of deep excavation deformation before inversion during Case 1.

To evaluate the simulation accuracy of the finite element model with soil parameters from site investigation and design, a finite element model before inversion is established based on the Wuhan Zhongyi Road Metro Station deep excavation project. With the progression of deep excavation, the cloud diagram of deep excavation deformation before inversion during Case 1 is presented in Figure 7. The deformation of the retaining structures is characterized by a larger magnitude in the middle and smaller magnitudes at both sides, which is consistent with the monitoring data variation trend.

**Figure 8:**
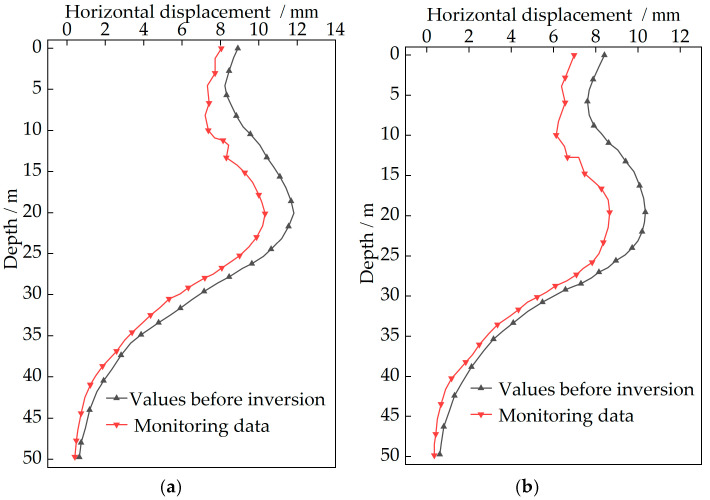
Comparison between simulated values before inversion and monitoring data during Case 1: (**a**) CX1 monitoring point; (**b**) CX2 monitoring point.

The construction stage of Case 1 is selected as the validation period for simulations due to pronounced displacement variations during this stage. Comparison between finite element simulated values before inversion at monitoring points CX1 and CX2 with monitoring data is presented in Figure 8.

As shown in Figure 8, the deformation of the upper retaining structures is constrained to some extent, while an increase in horizontal displacement is observed in the lower excavated section. At monitoring point CX1, the maximum monitoring displacement is recorded at 20.09 m with a value of 10.32 mm, and the simulated maximum value at the same depth is determined as 11.82 mm. At monitoring point CX2, the maximum monitoring displacement is observed at 19.57 m with a value of 8.65 mm, and the simulated maximum value at the same depth is calculated as 10.29 mm. The deformation pattern in the finite element simulation is essentially consistent with the monitoring data. It is demonstrated that the finite element simulation can authentically reflect the actual deep excavation process. However, certain numerical discrepancies are noted. It is exactly the inversion of soil parameters that is considered essential, since the accuracy of the finite element model is significantly affected by the soil parameters. Although the excavation process of the deep foundation pit can be realistically reflected by finite element simulation, numerical discrepancies still exist. Therefore, the soil parameters are determined based on the measured horizontal displacements of the retaining structure.

### 3.4. Sample Construction and Soil Parameters Inversion

Study [38,39] have demonstrated that the elastic modulus *E*, cohesion $C_{s}$, and internal friction angle φ exert the most significant influence on the horizontal displacements of deep excavation retaining structures. Therefore, the soil parameters of the undisturbed fill soil layer ($E_{1}$, $C_{s1}$, $\varphi_{1}$), clay layer ($E_{2}$, $C_{s2}$, $\varphi_{2}$), and silt layer ($E_{3}$, $C_{s3}$, $\varphi_{3}$) are selected as the inversion targets. Focusing the inversion parameters on these three most critical ones is a form of parameter screening based on prior knowledge. Based on the site investigation results, parameter constraints, which permit a variation within ±20%, serve as a regularization method based on prior knowledge. The value ranges of the soil parameters are listed in Table 2.

To reduce the computational complexity of finite element simulations, orthogonal testing is adopted for the selection of representative combinations of soil parameters. A total of 27 experimental sets is designed. Three parameters from three soil layers are selected for inversion, with three levels assigned to each parameter. The orthogonal design matrix is listed in Table 3.

The 27 sets of soil parameter combinations from the orthogonal design are sequentially input into the finite element model. A wider parameter range could be explored in the future when larger datasets are available. Horizontal displacement results of the retaining structures at critical depths of monitoring point CX1 are designated as J1 through J9. Sample test results are listed in Table 4.

The horizontal displacements of retaining structures obtained from finite element simulation and the soil parameters designed by orthogonal design are utilized as training samples. These training samples are randomly divided into a training set (22 groups) and a testing set (5 groups). The particle fitness of the SVM model under different parameter combinations is evaluated by the PSO algorithm using 5-fold cross-validation on the training set (22 groups). This internal cross-validation process prevents overfitting and ensures the robustness of the selected hyperparameters. The separate testing set (five groups) is strictly reserved for the final evaluation of the model generalization performance. The PSO algorithm is employed with the training and testing sets for iteration. The randomly initialized optimal penalty parameter *C* and kernel function parameter *g* for the SVM model under different parameter combinations are selected, and an optimized SVM model is obtained. The deep horizontal displacements of retaining structures at the critical depth of monitoring point CX1 are input. Corresponding inverted values of soil mechanical parameters are acquired. The inverted parameters correction range are compared with pre-inversion investigation values. The comparative analysis results are listed in Table 5. Compared with the investigation results, the correction range of elastic modulus *E* through inversion is corrected as −2.0% to 7.4%. The correction $C_{s}$ range of cohesion is corrected as −7.3% to 6.7%, while that of internal friction angle φ is corrected as −9.9% to 7.4%. It is demonstrated that correction ranges vary for different parameters across distinct soil layers, indicating differential sensitivity of soil parameters to the horizontal displacements of retaining structures. It is important to note that such notable changes may also arise from multiple sources, including missing data, potential influences from factors such as rainfall, as well as construction activities. Moreover, the significant correction ranges confirm the necessity for inversion of soil parameters in deep excavations.

### 3.5. Establishment of Inversion Finite Element Model for Deep Excavation

**Figure 9:**
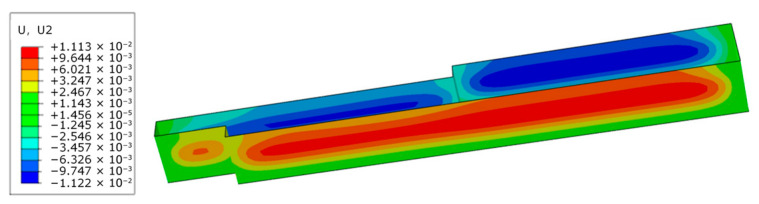
The deformation cloud diagram after inversion.

**Figure 10:**
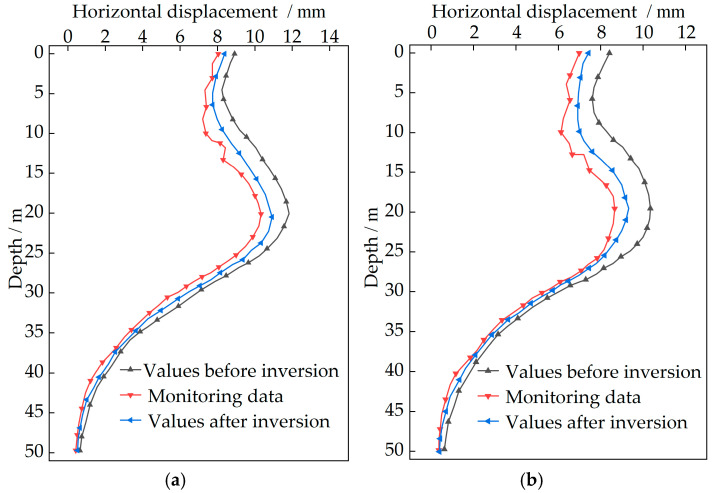
Comparison between simulated values before and after inversion and monitoring data during Case 1: (**a**) CX1 monitoring point; (**b**) CX2 monitoring point.

To verify the reliability of the inversion results, the accuracy of soil parameter inversion is validated by all monitoring data at different depths of monitoring point CX1 during Case 1. Simultaneously, to enhance the practical applicability of inversion, the horizontal displacements of retaining structures at monitoring point CX2 are calculated by the finite element model, further confirming the accuracy of soil parameter inversion. The deformation cloud diagram after inversion for Case 1 is presented in Figure 9. The simulated values before and after inversion at monitoring points CX1 and CX2 during Case 1 are compared with the monitoring data. The results are shown in Figure 10. A comparison of the maximum relative errors before and after inversion is listed in Table 6.

As indicated in Figure 10 and listed in Table 6, after inversion at CX1, the maximum simulated value is reduced from 11.82 mm to 10.91 mm, and the maximum relative error is decreased from 14.53% to 5.72%. At CX2, the maximum simulated value is lowered from 10.29 mm to 9.31 mm after inversion, with the maximum relative error reduced from 18.96% to 7.63%. It is concluded that finite element simulation results generally exceed monitoring values, with a maximum relative error reaching 18.96%. In contrast, simulation results after inversion demonstrate greater consistency with monitoring trends and smaller errors. The mechanical characteristics of the soil and deformation features during excavation are reflected more authentically. The model exhibits excellent practical applicability, confirming the high accuracy of inverted soil parameters for the deep excavation.

### 3.6. Prediction of Retaining Structure Deformation During Subsequent Cases

**Figure 11:**
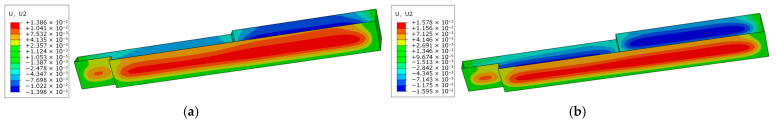
The deformation cloud diagrams of retaining structures after inversion: (**a**) deformation cloud diagram of Case 2; (**b**) deformation cloud diagram of Case 3.

**Figure 12:**
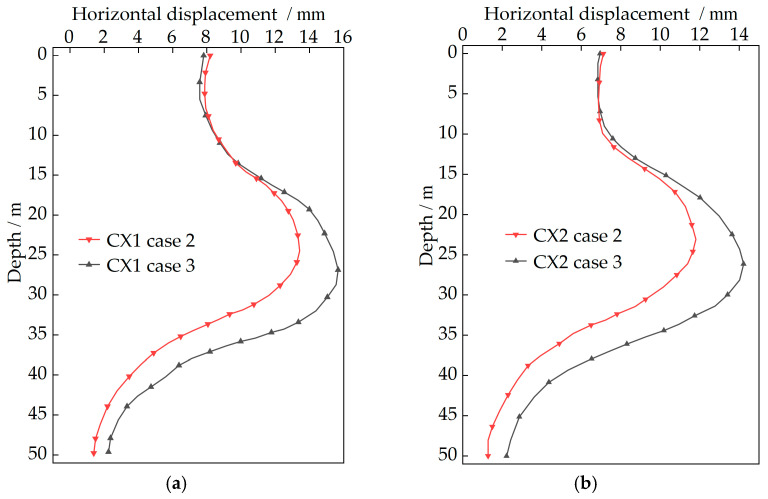
The predicted results of horizontal displacements for retaining structures: (**a**) prediction of CX1 monitoring point; (**b**) prediction of CX2 monitoring point.

The inverted soil parameters of the deep excavation are utilized to predict the horizontal displacements of retaining structures during subsequent Cases 2 and 3. The deformation cloud diagrams of retaining structures after inversion during these cases are presented in Figure 11. From the cloud diagrams, it is clearly observed that deformation is enlarged with increased excavation depth as construction progresses. The predicted results of horizontal displacements for retaining structures during excavation of the B2 level (Case 2) and B3 level (Case 3), as simulated by the finite element method, are shown in Figure 12. It is evident that the deformation range of horizontal displacement gradually shifts downward with greater excavation depth, while deformation magnitude increases concurrently. Maximum displacements at different monitoring points during subsequent cases are listed in Table 7. During Case 2, the maximum horizontal displacement at monitoring point CX1 is recorded as 13.42 mm at 24.48 m below pit top. During Case 3, this value reaches 15.66 mm at 26.85 m below pit top. At monitoring point CX2, the maximum horizontal displacement is monitored as 11.80 mm at 23.06 m below pit top during Case 2, and 14.22 mm at 26.15 m below pit top during Case 3. All deformation values remain below the project warning threshold of 30.00 mm [40].

## 4. Conclusions

To address the challenges of soil parameter variation and deformation prediction in numerical simulations for deep excavation engineering, a soil parameters inversion method based on a PSO-SVM model is proposed, with the Wuhan Zhongyi Road Metro Station deep excavation as the engineering case. The inverted soil parameters are acquired, and the accuracy of the inversion is verified. Subsequently, the horizontal displacements (deformation) of retaining structures during subsequent construction stages are predicted and analyzed. Conclusions are drawn as follows:

1. Based on orthogonal experimental design and finite element simulation, training samples are obtained. Optimal parameters for the PSO-SVM model are determined, and an optimal mapping relationship between the mechanical parameters of deep excavation soils and the horizontal displacements of retaining structures is established. A soil parameter inversion method for deep excavations is formed.
2. The accuracy of the finite element model is validated by monitoring data from the Wuhan Zhongyi Road Metro Station deep excavation. The soil parameter inversion can be performed with the optimized PSO-SVM model, and the deformation simulation results of retaining structures demonstrate greater consistency with monitoring trends. At monitoring point CX1, the maximum relative error of simulated horizontal displacement can be reduced from 14.53% to 5.72%. At CX2, this error can be decreased from 18.96% to 7.63%. These results indicate smaller errors and significantly improved accuracy for finite element simulations with inverted soil parameters.
3. During Case 2, the maximum horizontal displacements at CX1 and CX2 are recorded as 13.42 mm and 11.80 mm, respectively. During Case 3, these values reach 15.66 mm and 14.22 mm, respectively. All values remain below the project warning threshold of 30.00 mm. The practical applicability and safety reliability of the model are confirmed, which provides a reference for deep excavation analysis and evaluation during the construction period.

This study proposes a soil parameter inversion method based on the PSO-SVM model, which enables efficient and accurate inversion of soil parameters and allows for the prediction of subsequent working conditions. Its applicability under different geological conditions still requires further verification through more diverse engineering cases. In addition, the inversion accuracy highly depends on the quality and completeness of the monitoring data. Insufficient monitoring points or data gaps can significantly compromise the reliability of the results.

## Figures and Tables

**Table 1:** Soil parameters.

Thickness *h* (m)	Soil Layer	Severe *γ* (kN/m^3^)	Modulus of Elasticity *E* (MPa)	Poisson Ratio μ	Cohesion $C_{s}$ (kPa)	Internal Friction Angle φ (°)
1.40	Mixed fill	0	5.40	0.35	5.00	19.0
1.80	Fill soil	18.20	7.00	0.34	9.00	7.00
5.60	Clay	18.80	12.00	0.25	18.00	11.00
24.90	Silt	19.00	27.00	0.32	12.00	12.00
14.20	Pebble	20.00	114.00	0.28	0	35.00
6.20	Strongly weathered silty mudstone	23.50	215.00	0.27	111.00	26.00
25.90	Moderately weathered silty mudstone	24.80	260.00	0.24	220.00	31.00

**Table 2:** The value ranges of soil parameters.

**Horizontal**	$E_{1}$ (MPa)	$C_{s1}$ (kPa)	$\varphi_{1}$ (°)	$E_{2}$ (MPa)	$C_{s2}$ (kPa)	$\varphi_{2}$ (°)	$E_{3}$ (MPa)	$C_{s3}$ (kPa)	$\varphi_{3}$ (°)
1	5.60	7.20	5.60	9.60	14.40	8.80	21.60	9.60	9.60
2	7.00	9.00	7.00	12.00	18.00	11.00	27.00	12.00	12.00
3	8.40	10.80	8.40	14.40	21.60	13.20	32.40	14.40	14.40

**Table 3:** The orthogonal design matrix.

**Experiment**	$E_{1}$ (MPa)	$C_{s1}$ (kPa)	$\varphi_{1}$ (°)	$E_{2}$ (MPa)	$C_{s2}$ (kPa)	$\varphi_{2}$ (°)	$E_{3}$ (MPa)	$C_{s3}$ (kPa)	$\varphi_{3}$ (°)
1	5.60	7.20	5.60	9.60	14.40	8.80	21.60	9.60	9.60
2	5.60	7.20	5.60	9.60	18.00	11.00	27.00	12.00	12.00
3	5.60	7.20	5.60	9.60	21.60	13.20	32.40	14.40	14.40
4	5.60	9.00	7.00	12.00	14.40	8.80	21.60	12.00	12.00
5	5.60	9.00	7.00	12.00	18.00	11.00	27.00	14.40	14.40
6	5.60	9.00	7.00	12.00	21.60	13.20	32.40	9.60	9.60
7	5.60	10.80	8.40	14.40	14.40	8.80	21.60	14.40	14.40
8	5.60	10.80	8.40	14.40	18.00	11.00	27.00	9.60	9.60
9	5.60	10.80	8.40	14.40	21.60	13.20	32.40	12.00	12.00
10	7.00	7.20	5.60	14.40	14.40	11.00	32.40	9.60	12.00
11	7.00	7.20	7.00	14.40	18.00	13.20	21.60	12.00	14.40
12	7.00	7.20	8.40	14.40	21.60	8.80	27.00	14.40	9.60
13	7.00	9.00	5.60	9.60	14.40	11.00	32.40	12.00	14.40
14	7.00	9.00	7.00	9.60	18.00	13.20	21.60	14.40	9.60
15	7.00	9.00	8.40	9.60	21.60	8.80	27.00	9.60	12.00
16	7.00	10.80	5.60	12.00	14.40	11.00	32.40	14.40	9.60
17	7.00	10.80	7.00	12.00	18.00	13.20	21.60	9.60	12.00
18	7.00	10.80	8.40	12.00	21.60	8.80	27.00	12.00	14.40
19	8.40	7.20	8.40	12.00	14.40	13.20	27.00	9.60	14.40
20	8.40	7.20	8.40	12.00	18.00	8.80	32.40	12.00	9.60
21	8.40	7.20	8.40	12.00	21.60	11.00	21.60	14.40	12.00
22	8.40	9.00	5.60	14.40	14.40	13.20	27.00	12.00	9.60
23	8.40	9.00	5.60	14.40	18.00	8.80	32.40	14.40	12.00
24	8.40	9.00	5.60	14.40	21.60	11.00	21.60	9.60	14.40
25	8.40	10.80	7.00	9.60	14.40	13.20	27.00	14.40	12.00
26	8.40	10.80	7.00	9.60	18.00	8.80	32.40	9.60	14.40
27	8.40	10.80	7.00	9.60	21.60	11.00	21.60	12.00	9.60

**Table 4:** Horizontal displacement results of retaining structures for each test (mm).

Experiment	J1	J2	J3	J4	J5	J6	J7	J8	J9
1	7.79	6.67	6.58	7.65	9.01	10.24	11.39	8.92	8.56
2	8.45	7.75	7.63	8.72	9.20	11.07	11.23	10.11	8.06
3	7.87	7.65	6.76	8.89	8.92	10.04	10.78	10.05	8.13
4	7.67	7.16	6.81	8.68	9.11	10.24	10.93	9.11	7.61
5	8.70	7.31	7.18	8.03	10.21	10.48	11.41	10.01	8.44
6	8.91	7.25	7.12	8.22	8.64	10.06	10.86	9.16	7.10
7	8.26	8.07	7.35	8.14	8.51	9.65	9.71	9.24	8.31
8	8.01	6.82	6.77	9.05	9.50	11.31	10.46	10.23	9.31
9	8.69	7.71	7.25	8.86	9.61	9.94	11.47	10.16	8.50
10	8.87	7.80	7.49	8.98	9.69	10.24	10.93	10.65	8.90
11	8.31	7.88	6.43	7.59	9.44	10.79	11.20	9.87	8.54
12	7.83	7.41	6.46	8.14	8.58	9.88	11.39	9.28	8.75
13	8.27	8.13	7.59	8.62	9.13	10.01	10.49	9.62	7.66
14	7.59	7.31	6.68	8.63	9.12	10.95	10.16	8.21	7.69
15	8.27	7.75	7.25	7.49	8.14	9.14	10.98	9.47	8.77
16	8.47	7.83	7.13	9.06	9.58	9.65	10.31	9.51	8.52
17	8.75	7.74	6.80	8.62	9.78	10.84	11.43	8.71	8.40
18	7.78	7.21	6.51	8.73	9.12	9.60	9.72	8.54	7.62
19	7.79	7.54	7.26	8.45	8.83	9.44	9.01	8.23	7.81
20	8.16	7.72	7.03	8.43	8.54	9.96	10.10	9.04	8.12
21	9.27	8.21	7.11	8.34	9.14	10.57	11.74	10.45	9.04
22	8.33	7.36	6.98	8.48	9.23	10.13	10.82	9.23	8.50
23	7.83	7.30	7.21	8.85	8.29	8.99	9.15	8.66	7.44
24	8.01	7.88	7.43	8.41	9.25	9.65	10.33	8.59	7.04
25	8.23	7.79	7.06	8.42	9.18	10.08	10.64	9.76	8.48
26	7.60	6.68	6.46	8.12	8.43	9.55	9.87	8.95	7.35
27	8.07	7.76	7.21	8.28	9.80	10.28	10.41	8.66	7.42

**Table 5:** The comparative analysis results of soil parameters correction range.

**Horizontal**	$E_{1}$ (MPa)	$C_{s1}$ (kPa)	$\varphi_{1}$ (°)	$E_{2}$ (MPa)	$C_{s2}$ (kPa)	$\varphi_{2}$ (°)	$E_{3}$ (MPa)	$C_{s3}$ (kPa)	$\varphi_{3}$ (°)
Before inversion	7.00	9.00	7.00	12.00	18.00	11.00	27.00	12.00	12.00
After inversion	7.52	9.43	6.31	11.76	19.21	11.34	27.54	11.12	12.89
Correction range	7.40%	4.80%	−9.90%	−2.00%	6.70%	3.10%	2.00%	−7.30%	7.40%

**Table 6:** Comparison of maximum relative errors before and after inversion.

Monitoring Point		Before Inversion			After Inversion		
	Horizontal Displacement	Maximum Horizontal Displacement/mm	Absolute Error/mm	Relative Error/%	Maximum Horizontal Displacement/mm	Absolute Error/mm	Relative Error/%
CX1	10.32	11.82	1.50	14.53%	10.91	0.59	5.72%
CX2	8.65	10.29	1.64	18.96%	9.31	0.66	7.63%

**Table 7:** Maximum horizontal displacement of different monitoring points during different cases.

Measuring Point	Case	Depth of Maximum Horizontal Displacement	Maximum Horizontal Displacement
CX1	2	24.48 m	13.42 mm
	3	26.85 m	15.66 mm
CX2	2	23.06 m	11.80 mm
	3	26.15 m	14.22 mm

## Data Availability

The data presented in this study are available at the request from the corresponding author.

## Abbreviations

The following abbreviations are used in this manuscript:

BP	Back Propagation Neural Network
CNN	Convolutional Neural Network
LSTM	Long Short-Term Memory
SVM	Support Vector Machine
PSO	Particle Swarm Optimization
